# The Mildew Resistance Locus O 4 Interacts with CaM/CML and Is Involved in Root Gravity Response

**DOI:** 10.3390/ijms22115962

**Published:** 2021-05-31

**Authors:** Lei Zhu, Xue-Qin Zhang, De Ye, Li-Qun Chen

**Affiliations:** 1 College of Biological Sciences, China Agricultural University, Beijing 100193, China; kouyang@cau.edu.cn (X.-Q.Z.); yede@cau.edu.cn (D.Y.); 2 Beijing Advanced Innovation Center for Tree Breeding by Molecular Design, Beijing Forestry University, Beijing 100083, China

**Keywords:** calmodulin, calmodulin-like proteins, CML12, MLO protein, MLO4, root gravitropism

## Abstract

The plant-specific mildew resistance locus O (MLO) proteins, which contain seven transmembrane domains and a conserved calmodulin-binding domain, play important roles in many plant developmental processes. However, their mechanisms that regulate plant development remain unclear. Here, we report the functional characterization of the MLO4 protein in *Arabidopsis* roots. The MLO4 was identified as interacting with CML12 in a screening for the interaction between the proteins from *Arabidopsis* MLO and calmodulin/calmodulin-like (CaM/CML) families using yeast two hybrid (Y2H) assays. Then, the interaction between MLO4 and CML12 was further verified by Luciferase Complementation Imaging (LCI) and Bimolecular Fluorescence Complementation (BiFC) assays. Genetic analysis showed that the *mlo4*, *cml12,* and *mlo4 cml12* mutants displayed similar defects in root gravity response. These results imply that the MLO4 might play an important role in root gravity response through interaction with CML12. Moreover, our results also demonstrated that the interaction between the MLO and CaM/CML families might be conservative.

## 1. Introduction

Mildew resistance locus O (MLO) was first identified as being resistant against powdery mildew (PM) infection in barley [1]. Then, the MLO proteins were found in many plant species. *Arabidopsis* has at least 15 MLO proteins that diverged into several clades and were involved in different physiological processes, such as sexual reproduction, PM infection, and root thigmomorphogenesis [2,3,4,5]. MLO7/NORTIA (NTA), a downstream component of the receptor-like kinase FERONIA (FER), is involved in pollen tube reception and PM infection [6]. Study showed that its function is dependent on NTA homooligomerization and its carboxyl-terminal tail identity [7]. MLO5/9/15 played roles in pollen tube responses to ovular signals, and MLO5/9 selectively recruit Ca^2+^ channel CNGC18-containing vesicles to the plasma membrane [8]. The MLO1, MLO2, MLO3, MLO6, MLO8, MLO9, MLO10, and MLO13 are expressed in discrete domains during reproductive development [4]. The MLO2, MLO4, and MLO6 modulate defense responses against PM fungi and a number of other phytopathogens [9]. Furthermore, the MLO2 also function as a negative regulator in plant ROS responses involving biotic and abiotic stress [10]. Mutations in *MLO4* and *MLO11* exhibit abnormal root thigmomorphogenesis and gravity sensitivity [3,11]. Genetical complementation with MLO4 domains indicate that the C-terminal cytoplasmic domain of MLO4 is necessary for regulation of asymmetrical root growth [11].

All MLO proteins are predicted to share a conserved calmodulin-binding domain (CaMBD) in C-terminal cytoplasmic tail [12,13]. The gel overlay assays demonstrated in vitro Ca^2+^-dependent binding of CaM to the CaMBD of the MLOs from barley [14]. However, little is known about the interaction of individual MLO with the specific calmodulins (CaMs) or calmodulin-like proteins (CML) in *Arabidopsis* or other plants.

The *Arabidopsis* genome encodes 7 CaMs and 50 CMLs, which are presumed to sense and transduce Ca^2+^ signals [15]. Functional studies of CaM/CML in plants revealed that this protein family converts calcium signals into transcriptional responses, protein phosphorylation, or metabolic changes to regulate plant development responding to the ever-changing environment [16]. Some CaM/CML proteins are involved in plant growth and development, such as AtCaM7 regulating light-induced seedling development [17,18], AtCaM2 functioning in pollen germination [19], AtCML42 for trichome morphogenesis [20], and AtCML23 and AtCML24 in flowering [21,22]. Some CaM/CML proteins are indispensable for plants to respond to abiotic and biotic stress. The mutations in *AtCaM3* lead to reduction in thermotolerance of the mutant plants [23]. AtCaM4 negatively regulates freezing tolerance by interacting with CaM-binding protein PATL1 in a CBF-independent manner [24]. A knockout mutant of *AtCML9* enhances its tolerance to drought and salinity stress [25]. AtCML20 is a negative regulator in guard cell ABA signaling during drought tolerance [26]. AtCML8 and AtCML9 positively regulate plant immunity in response to *Pst* inoculation [27]. Less NtCaM13 expression causes more susceptibility to virulent bacteria and fungi in tobacco [28]. Overexpression of pepper CaM1 and AtCML43 confers enhanced resistance to pathogens [29]. All these data suggest that the CaM/CML-mediated defense signaling pathways involve a complex regulatory network.

Both MLO and CaM/CML proteins are important for plants. They have potential to interact with each other in between the two families through CaMBD [12]. However, for now, it is just predicted by physical structure of the proteins, and not solid evidence, to prove their interaction or even their related biological function.

Our goals in this study were to preliminarily grasp the co-expression patterns of *MLO* and *CaM/CML* genes, to screen the interactive protein pairs between MLO and CaM/CML family, and to investigate the biological function of the interactive MLO and CaM/CML proteins. In our results, *MLO* and *CaM/CML* genes were expressed in various tissues and functioned in different developmental stages. Each MLO protein interacted with more than one CaM/CML protein by Y2H screen. Two groups of interactive MLO and CaM/CML proteins, MLO4 and CML12; MLO7/NTA and CML8-12, were further confirmed by BiFC and LCI assays. Moreover, the biological function analysis showed that the *mlo4* and *cml12* single mutants, as well as the *mlo4 cml12* double mutant, shared similar phenotypes in root growth. These results indicate that MLO4 is involved in root gravity response, possibly through interaction with CaM/CML protein.

## 2. Results

### 2.1. The Expression Patterns of MLO and CaM/CML Genes

**Figure 1:**
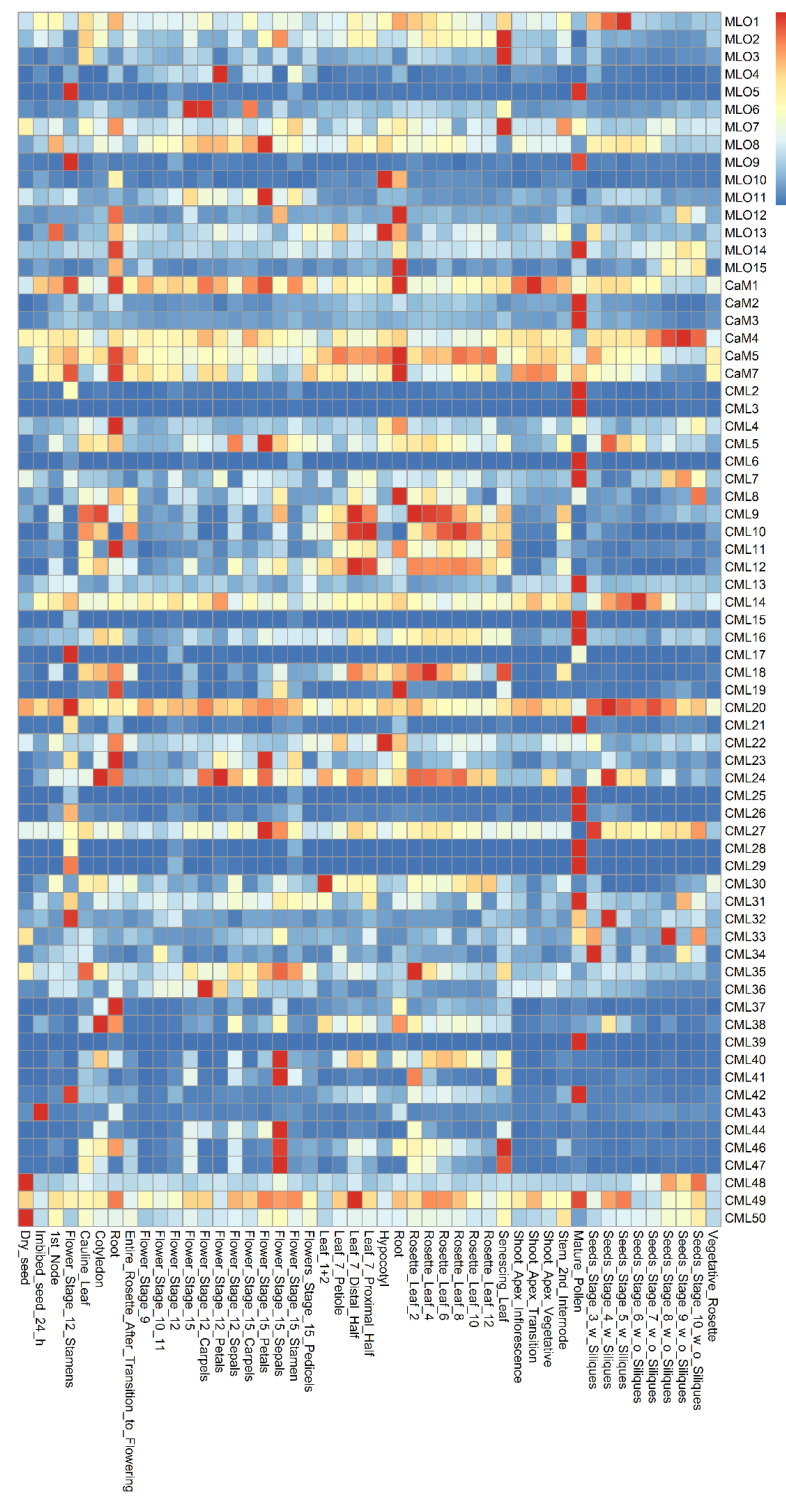
Expression pattern of *Arabidopsis* MLO and CaM/CML family genes.

The co-expression patterns of *MLO* and *CaM/CML* genes were analyzed using the e-FP data from TAIR website (www.arabidopsis.org, accessed on 19 May 2021) (Figure 1). The results showed that the *MLO* and *CaM/CML* genes are expressed in various *Arabidopsis* tissues. The *MLO1* was expressed more highly in seeds than in other tissues. The *CaM*/*CML* genes, which exhibit similar expression in seeds, included 12 members, namely, *CaM4*, *CML14*, *CML20*, *CML24*, *CML27*, *CML32*, *CML33*, *CML34*, *CML43*, *CML48*, *CML49,* and *CML50*. The *MLO5*, *MLO9,* and *MLO14* genes were highly expressed in pollen. In the pollen, 17 *CaM*/*CML* genes, including *CaM2*, *CaM3*, *CML2*, *CML3*, *CML6*, *CML7*, *CML13*, *CML15*, *CML16*, *CML21*, *CML26*, *CML28*, *CML29*, *CML31*, *CML39*, *CML42,* and *CML49*, were found to be highly co-expressed. In flowers, four *MLO* genes (*MLO4*, *MLO6*, *MLO8,* and *MLO11*) and nine *CML* genes (*CML5*, *CML24*, *CML27*, *CML36*, *CML40*, *CML41*, *CML44*, *CML46,* and *CML47*) were highly co-expressed. The four *MLO* genes, *MLO2*, *MLO3,* and *MLO8*, were found to be highly expressed in leaves; similarly, five *CML* genes were highly expressed in leaves, namely, *CML9*, *CML10*, *CML18*, *CML35,* and *CML46*. In roots, *MLO12* gene was highly co-expressed, together with five *CaM/CML* genes (*CaM1*, *CaM5*, *CaM7*, *CML8,* and *CML19*). *MLO4* and *CML12* were expressed in many tissues, including roots. Although the highest expression levels of both *MLO4* and *CML12* did not present in roots, they were highly co-expressed in roots, implying both of them may function in roots.

### 2.2. Screening for Interactive Pairs of MLO and CaM/CML Proteins

The MLO proteins contain a CaMBD in the C-terminus region [12]. Therefore, the C-terminus regions of the 15 MLO proteins were used to characterize the interaction of MLO and CaM/CML proteins by Y2H. The results showed that each MLO protein may interact with at least one CML protein in yeast cells (Table 1, Tables S1 and S2). In particular, the NTA/MLO7, which was involved in pollen tube reception, could interact with up to 11 candidate CML proteins, including CML8-12, CML26, CML30, CML37, CML38, CML40, and CML40. Similar results were also obtained from the assays for the three closely-related proteins MLO4, MLO11, and MLO14, which function in root thigmomorphogenesis [11]. MLO4 interacted with CML12, CML40, and CML44. MLO11 interacted with CML47. MLO14 interacted with CML17, CML18, and CML49.

### 2.3. LCI and BiFC Assays Further Demonstrated That MLO4 Interacted with CML12

**Figure 2:**
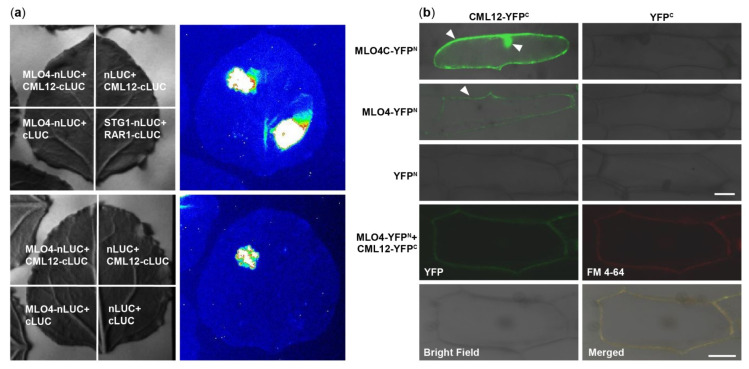
CML12 interacted with MLO4. (**a**) LCI results showed CML12 interacted with full length of MLO4 in tobacco cells. (**b**) BiFC results showed that CML12 interacted with cytoplasmic domain of MLO4 in onion cells, and the signal was detected in nucleus and periphery. When CML12 interacted with full length of MLO4, the signal was only detected on the plasma membrane, colocalized with FM 4-64. The YFP signals are indicated by pointed triangles in each figure. Bars = 50 μm.

At first, MLO4 and CML12 were chosen to verify the results of Y2H screening (Figure S1). Then, the LCI results showed that CML12 interacted with the full length of MLO4 in tobacco cells (Figure 2a). BiFC results demonstrated that the CML12 interacted with the cytoplasmic domain of MLO4, in concert to Y2H result. Furthermore, when the full length of MLO4 was used for the assays, the interactive signal was detected only on the plasma membrane, which was co-localized with the staining signals of the membrane dye FM 4-64 (Figure 2b). This result was consistent to the ones obtained from the membrane localization of MLO4 with a signal peptide and seven transmembrane domains [11].

### 2.4. MLO4 and CML12 Displayed a Similar Expression Pattern

**Figure 3:**
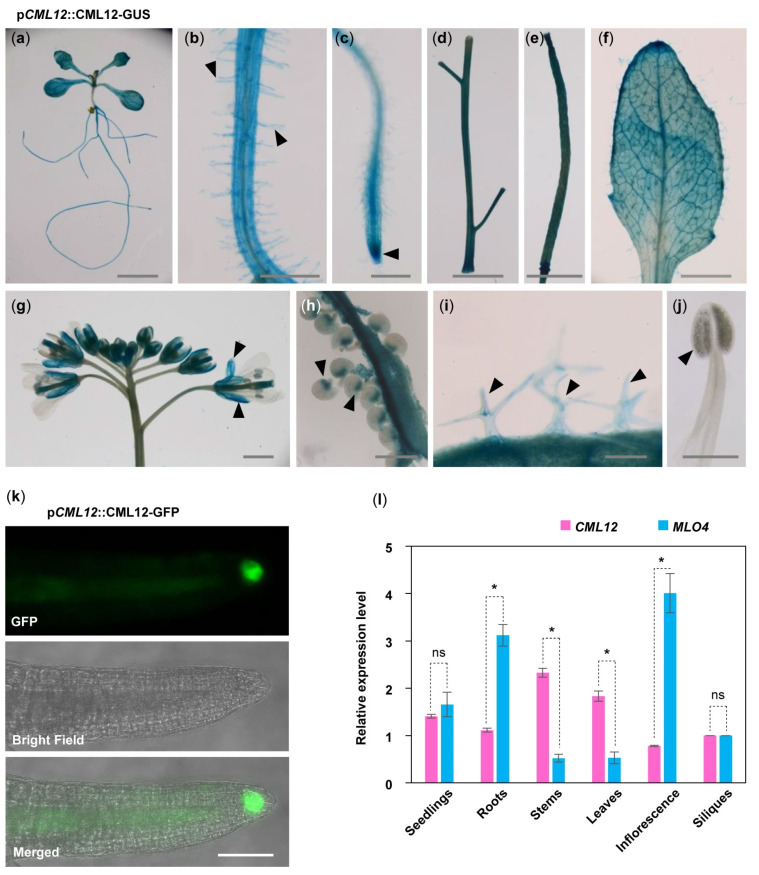
Expression pattern of *CML12* Gene. GUS staining of p*CML12::CML12-GUS* transgenetic lines showed *CML12* was expressed in most of the vegetable tissues: Seedling (**a**), root hairs (**b**), root tip (**c**), stems (**d**), silique (**e**), leaf (**f**), sepals (**g**), chalazal end of ovules (**h**), and trichomes (**i**), not in pollen (**j**), as indicated by pointed triangles in each figure. Bars = 100 μm. (**k**) p*CML12::CML12-GFP* transgenetic lines showed CML12-GFP was detected in root cap. Bar = 20 μm. (**l**) Real-time PCR showed that *CML12* and *MLO4* genes both were expressed in various tissues of *Arabidopsis*. ns: not significant; the * above bars indicate the *t*-test results at significant level of 0.05.

To understand the relationship between *MLO4* and *CML12*, we further investigated whether *MLO4* and *CML12* have a co-expression pattern in vivo. First, GUS staining of p*CML12::CML12-GUS* transgenic lines showed that *CML12* was expressed in most of the vegetable tissues, such as seedlings, roots, root hairs, leaves, trichomes, stems, siliques, calyxes, and chalazal end of ovules but not in pollen (Figure 3a–j). In addition, p*CML12::CML12-GFP* transgenic lines showed that the CML12-GFP fusion protein was detected in the root cap (Figure 3k). Real-time PCR results showed that *MLO4* and *CML12* shared a similar expression pattern. Both genes were expressed constitutively in many tissues, including seedlings, roots, stems, leaves, inflorescence, and siliques. Expression levels of both genes were closely similar in seedlings. *MLO4* was expressed with twice the *CML12* expression in roots (Figure 3l). A previous study revealed that the p*MLO4::MLO4-GFP* was also expressed in the roots, and MLO4-GFP fusion protein was detected in the root epidermal cell [11]. Therefore, both CML12 and MLO4 proteins were expressed in roots, suggesting that they may function coordinately.

### 2.5. The mlo4 and cml12 Mutants Shared Similar Phenotypes

The MLO4 has been characterized as regulating root thigmomorphogenesis, including gravitropism in roots [11]. Previous characterization of *cml12* revealed that the mutations in *CML12* exhibited a similar phenotype [30]. To study the genetic relationship between *MLO4* and *CML12*, we further characterized their roles in root growth.

**Figure 4:**
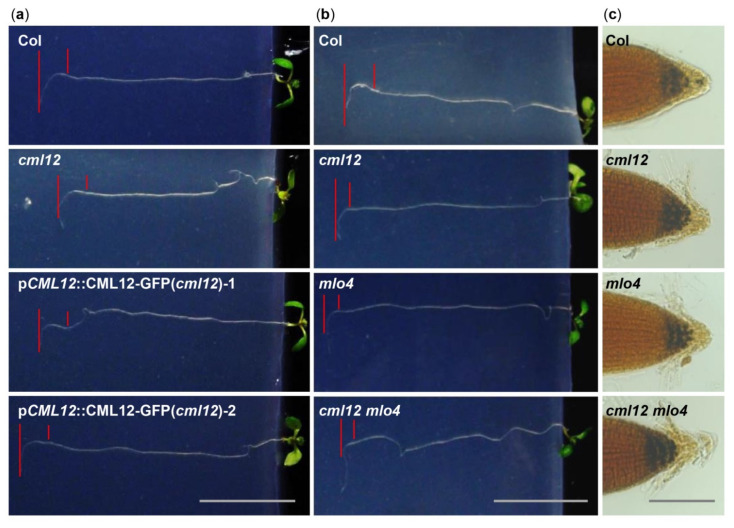
Phenotype analysis of *cml12-3* and *mlo4-5* mutants. *cml12-3* and *mlo4-5* mutants were indicated, respectively, as *cml12* and *mlo4,* for short. (**a**) 7-day-old seedlings growing through the agar medium were rotated by 90° in the clockwise direction. Roots bent down to respond to gravity change. Red lines point to tip position at the time of rotation and the location where root growth became parallel with new gravity vector. Bar = 1 cm. (**b**) Roots bent down to respond to gravity change. Red lines point to tip position at the time of rotation and the location where root growth became parallel with new gravity vector. Bar = 1 cm. (**c**) Starch was detected using KI-I_2_ stain Starch in single mutants *cml12-3* and *mlo4-5* and double mutant *cml12-3 mlo4-5* was obviously more than that in wild-type root tips. Bar = 100 μm.

**Figure 5:**
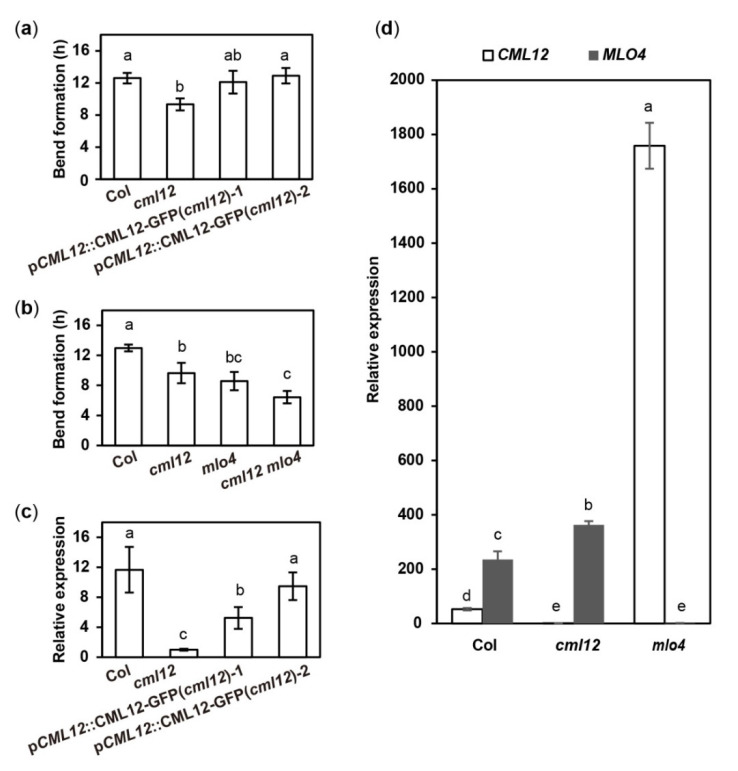
Quantitative analysis of bend formation and gene expression in *cml12-3* and *mlo4-5* mutants. *cml12-3* and *mlo4-5* mutants were indicated, respectively, as *cml12* and *mlo4* for short. (**a**)The time taken to form a bend in Col, *cml12-3*, p*CML12::CML12-GFP* (*cml12-3*)-1, and pCML12::CML12-GFP (*cml12-3*)-2 were 12.60 ± 0.66, 9.33 ± 0.75, 12.10 ± 1.42, and 12.90 ± 0.96 hours, respectively, 80 < *n* < 150. (**b**) The time taken to form a bend in Col, *cml12-3*, *mlo4-5,* and *cml12-3 mlo4-5* were 12.97 ± 0.45, 9.63 ± 1.36, 8.57 ± 1.22, and 6.43 ± 0.83 h, respectively, 80 < *n* < 150. (**c**) Real-time PCR showed that expression of *CML12* gene was restored in p*CML12::CML12-GFP* (*cml12-3/-*) plants. (**d**) Real-time PCR showed that relative expression of *CML12* increased significantly in *mlo4-5* mutant. Different letters above the bars indicate significant differences (*P* < 0.05, Bonferroni correction) in a pairwise comparison using the least significant difference (LSD) method.

The *cml12-3* mutant (SALK_122731) was obtained from Dr. Sherryl R. Bisgrove (Department of Biological Sciences, Simon Fraser University, Burnaby, BC, Canada). Real-time PCR showed that *cml12-3* was a knock-out mutant, as reported by Gleeson et al. [31] (Figure S2a,d). To test its gravity response, *cml12-3* seedlings were vertically grown on the agar minimal medium (0.25% sucrose) for 7 d (16 h light/8 h dark cycle); then, the plates were rotated by 90° in the clockwise direction. The roots bent down in response to gravity change until root tips became parallel with new gravity vector (Figure 4a). Compared to the time (12.60 ± 0.66 h) for the wild-type roots to form a bend, the *cml12-3* roots took less time (9.33 ± 0.75 h) to form a bend (Figure 5a), suggesting that *cml12-3* roots were more sensitive to gravity response.

To verify the phenotype of *cml12-3* in gravity response, a complementation assay was performed using the construct p*CML12::CML12-GFP* in PCAMBIA 1300 vector containing the native promoter (1500 bp upstream ATG) and coding sequence of *CML12* (975 bp). Two p*CML12::CML12-GFP* transgenic *cml12-3* mutant lines were chosen to further characterize their root phenotype. The average times for the roots from each p*CML12::CML12-GFP* (*cml12-3/-*) transgenic lines to form a bend were 12.10 ± 1.42 h and 12.90 ± 0.96 h, respectively, similar to that of wild-type roots (12.60 ± 0.66 h) (Figure 4a). The real-time PCR results showed that *CML12* expression was higher in transgenic roots than that in *cml12-3* mutant roots (Figure 5a). Therefore, *CML12* was able to restore the defect of *cml12-3* mutants in gravity response, further demonstrating that *CML12* is involved in gravity response.

The previous study showed that *mlo4-1*, *mlo4-3,* and *mlo4-4* mutants exhibited different growth patterns on the reclined medium than wild-type [11]. In this study, we identified another Salk allele *mlo4-5* in which the T-DNA was inserted in the last exon of *MLO4* (Figure S2b), different from the previously-reported alleles (Figure S2c). Real-time PCR showed that *mlo4-5* was a knock-out mutant and expressed truncated protein (Figure S2e). At first, an allelic analysis was performed. The F1 progeny of *mlo4-5* from the crosses with *mlo4-4* exhibited a resembled phenotype in growth pattern (Figure S3). The loops in each root of *mlo4-4/+; mlo4-5/+* plant were denser with shorter wavelengths, and its phenotype was similar to that of single mutant-*mlo4-4/-* and *mlo4-5/-*. The result demonstrated that *mlo4-5* also was an effective mutant of *MLO4* gene. Therefore, the *mlo4-5* was used for further characterization.

The phenotypic comparison in root gravity response was performed with the single mutant *cml12-3* (*cml12-3/-*) and *mlo4-5* (*mlo4-5/-*), as well as the double mutant *cml12-3 mlo4-5* (*cml12-3/-; mlo4-5/-*). The time for bend formation was 12.97 ± 0.45 h in wild-type roots. In contrast, the roots of the single mutant *cml12-3*, *mlo4-5* took 9.63 ± 0.45 h and 8.57 ± 1.22 h to form bends, respectively. The roots of the double mutant *cml12-3 mlo4-5* took relatively shorter time (6.43 ± 0.83 h) to form a bend (Figure 4b, Figure 5b), indicating that the phenotype of double mutant was a little stronger than those of the single mutants. 

Starch grains in root tips are sensors of gravity response [30]. Therefore, the starch stain assays were further performed to compare the starch contents in the mutants and wild-type root tips using KI-I_2_ staining. Contents of the starch grains in the single mutant *cml12-3* and *mlo4-5,* as well as the double mutant *cml12-3 mlo4-5,* were obviously higher than that in wild-type root tips (Figure 4c).

In addition, *cml12-3*, *mlo4-5,* and *cml12-3 mlo4-5* mutant roots shared similar growth patterns with denser loops and shorter wavelengths on the reclined agar medium. The phenotype of the double mutant *cml12-3 mlo4-5* was not obviously aggravated (Figure S4).

### 2.6. Mutation in MLO4 Affected the Expression of CML12

CaM/CMLs bound calcium and were supposed to regulate target proteins downstream [16]. To further study the possible regulatory relationship between *CML12* and *MLO4*, their relative expression levels in each other mutants were analyzed. The real-time PCR assay showed the expression of *CML12* was significantly elevated in *mlo4-5* mutant, far more than MLO4 expression changing in *cml12-3* mutant (Figure 5d). Thus, *MLO4* likely modulates the expression of *CML12*.

## 3. Discussion

### 3.1. MLO4 and CML12 Function Coordinately in Root Thigmomorphogenesis

In this study, the *cml12-3* roots were more sensitive to gravity, as demonstrated by taking less time for them to form a bend in gravity response assays. Moreover, the *CML12* gene could restore the *cml12-3* phenotype. These results suggest that *CML12* regulates root gravity response. The transgenic plants of p*CML12::CML12-GUS* and p*CML12::CML12-GFP* showed that CML12 protein was expressed in root, especially in root cap. Previous studies showed that gravity sensing happens in the root cap and is related to the starch sediment in the root cap [32,33,34,35,36]. Starch-filled amyloplasts sediment settled in the bottom of statocytes under gravity [35]. In starch-deficient mutants, amyloplasts did not precipitate and gravity response was deficient [37]. Characterization of both starch-less and starch-more mutants suggested that starch amount was directly associated with gravity sensing [30]. In *cml12-3* and *mlo4-5* mutants, starch grains detected by I_2_-KI stain were more than those in wild-type. Both mutants exhibited more sensitive gravity sensing than wild-type. Therefore, MLO4 and CML12 may be involved in gravity sensing through regulating starch content in root caps.

In the *mlo4-5* mutant, *CML12* expression was dramatically upregulated, implying that MLO4 may negatively modulate the expression of *CML12*. The *mlo4-5* and *cml12*-3mutants shared a similar growth pattern that exhibited a more sensitive response to gravity on the reclined medium plates in comparison with that of wild-type plant (Figure 4a and Figure 5a). Moreover, MLO4 and CML12 could interact with others. These results indicate that MLO4 and CML12 may function coordinately to regulate root thigmomorphogenesis. However, it is unclear how loss of *MLO4* could promote expression of *CML12*. *CML12*, also called *TOUCH3/TCH3*, is a touch-induced gene whose mRNA increases massively within seconds in response to various environmental stimuli, including touch, wind, and darkness [38]. A newly changed gravity vector may be a stimulus upregulating *CML12* expression, or there may be also other unknown regulators involved in regulation of *CML12* expression. Nevertheless, more studies are required to explain the regulatory mechanism of MLO4 and CML12. 

### 3.2. The Conservative Amino Acids of CaMBDs and C-terminal Sequence of MLO Protein May Determine Their Specific Interacting CaM/CMLs

MLO proteins are encoded by medium-sized gene families present in all investigated land plant species and they share a conserved CaMBD in cytoplasmic C-termini, which consists of 20 amino acids [39,40].

As reported previously, the barley MLO C-terminus interacted with soy bean CaM1. When the conservative amino acids (L420R, W423R) in CaMBD were mutated, the interaction of mutated HvMLO with SCaM1 was weak and even became undetectable [14]. These results suggest that the MLO–CML interaction is likely to depend on the specific CaMBD in C-terminus of MLO proteins. The C-terminal sequences of MLO proteins are obviously diversified. Studies showed that C-terminal regions of the MLO proteins could determine specific binding to the individual CaM/CML proteins to fulfill their distinct bio-function [13,41,42]. Domain-swap experiments testified that the identity of the C-terminal intracellular tail contributes to MLO function [43].

As confirmed in our study, the Y2H assays also showed that, for each MLO protein, only the C-terminus region was sufficient for binding the CaM/CML proteins (Table 1). Each MLO might interact with more than one CaM/CML proteins. The specific interaction between MLO and CaM/CML proteins are due to CaMBD of the MLO C-terminus. First, Y2H screen results revealed that C-terminus region of NTA/MLO7 interacted with CML8-12 (Figure S5). Then, Y2H assays was conducted with site mutation in CaMBD of C-terminus. NTA variants with modification of the conservative sites (L452R, W455R) could not interact with CML12, while NTA variants with changes in the non-conservative sites (K457N, L464R) remained able to interact with CML12. All NTA variants (L452R, W455R, K457N, L464R) failed to interact with CML8 or CML9 but still bound CML10 and CML11 (Figure S6).

Therefore, the invariant and conservative amino acids may enable CaMBD to bind CaM/CMLs. Meanwhile, the variant amino acids and C-terminal sequence may determine their specific interaction with CaM/CMLs, with the former directly affecting binding ability and the latter indirectly influencing MLO-CaM/CML spatial distance via conformation.

### 3.3. MLO-CaM/CML Interaction May Relate to Ca^2+^ Signaling and Mediate Vesicle Transport

The Ca^2+^ dynamics were detected in various cells upon responding to extracellular signals, for instance, the root development and root hair growth, the synergid cells sensing the arriving pollen tube, and vice versa, the pollen tube tips sensing ovules during sexual reproduction [8,44,45,46,47]. Meanwhile, more and more evidence has pointed out that vesicle transport was revealed in the corresponding root tips, synergid cells, and pollen tube tips [43,46,48].

The CaM/CML proteins distinguish themselves with EF-hands that can bind Ca^2+^, a second messenger generated by various stimulations [49]. Previous study has demonstrated that CML12 could bind to Ca^2+^ in vitro [38]. Additionally, nuclear Ca^2+^ signatures are detected in root cells and associated with root development [46,47]. MLO4 protein was located in vesicle membrane of root cells, and its subcellular localization responded to vesicle traffic inhibitor BFA. The expression of auxin efflux carrier fusion, PIN1-GFP, was altered at root tip of *mlo4* mutant seedlings, implying that MLO4 protein participated in auxin transport [11]. Now that MLO4 interacts with CML12 (Figure 2), this case provides the first scattered clues where Ca^2+^ signaling and vesicle transport may be related by MLO4–CML12 interaction.

The second case is Ca^2+^ oscillations displayed in the synergid cells and the FER-NTA pathway is required for Ca^2+^ responses [44,45,48]. NTA/MLO7 protein is located in vesicular-membrane and evenly distributed in synergid cells before arrival of the pollen tube. Upon pollen tube arrival, it is then polarly transferred to the micropylar end. In this way, MLO might deliver out certain signal through vesicle traffic by responding to the approaching pollen tube [6]. The interaction between NTA/MLO7 and CML8–12 was also verified by Y2H, BiFC, and LCI assays (Figures S5–S8), although later genetic analysis showed that the quadruple mutant *cml8 cml9 cml11 cml12* has normal fertility, not similar to that of the *nta/mlo7* mutant, indicating the possible functional redundancy of the CML proteins (CML10/26/30/37/38/40/44) (Table 1). Therefore, NTA/MLO7 protein may mediate Ca^2+^ signaling and vesicle transport by interacting with CMLs during pollen tube reception.

The third case is that Ca^2+^ formed a tip-focused gradient in the pollen tube and underwent oscillation in the tip region during pollen tube growth [8,43]. The pollen-specific MLO5/9 was distributed on vesicle membrane in the tip region of the pollen tube and directly bound Ca^2+^ channel CNGC18, which recruited CNGC18-containing vesicles to the plasma membrane through the R-SNARE proteins VAMP721 and VAMP722 in trans mode, in response to ovular signals [8]. In short, MLO5/9 mediated the exocytosis of a Ca^2+^ channel to reply to ovular guidance. In addition to that, our Y2H screen disclosed that MLO5 interacted with CML18/26/40/44, MLO9 interacted with CML10/42/44 (Table 1), which, of course, needs further verifying. Therefore, it is rational to deduce that MLO5/9-CML interaction may relate to Ca^2+^ signaling and mediate vesicle transport.

In conclusion, responding to extracellular signals, MLO proteins seem to modulate SNARE-dependent vesicle transport-associated processes at plasma membrane, in close relation to Ca^2+^ signaling, possibly through interacting with CaM/CMLs.

The MLO proteins are unique to plants [40]. Although some *mlo* mutants have been characterized, the mechanisms of the modulation of CaM by MLO proteins remain poorly understood. The biochemical function of MLO in modulating Ca^2+^-loaded CaM/CML also remains elusive.

## 4. Materials and Methods

### 4.1. Plant Materials and Growth Conditions

*Arabidopsis thaliana* plants in Columbia (Col) background were used in this study. The *cml12-3* (SALK_122731) was a gift from Dr. Sherryl R. Bisgrove [31]. The primer pairs used for the mutant characterization were LBa1 and SK122731-1 (Table S3). The *mlo4-4* (SAIL_266_C02) and *mlo4-5* (SAIL_563_E06) were purchased from the *Arabidopsis* Biological Resource Center (ABRC, www.arabidopsis.org, accessed on 19 May 2021). The primer pairs for identifying *mlo4-4* and *mlo4-5* were SAIL-LB3/SAIL266C02-1 and SAIL-LB3/SAIL563E06-1, respectively (Table S3). The seeds were surface-sterilized in the solution containing NaClO:ddH_2_O (1:8) for 5 minutes and washed with sterilized ddH_2_O three times. Then, the seeds were sowed on 1/2 MS culture medium and stood in 4 °C for 2–4 days. The vernalized seeds on 1/2 MS were cultured under a 16 h/8 h day/night cycle at 22 °C for 7–10 days for further growth. The resulting seedlings were then planted into soil under a 16 h/8 h day/night cycle at 22 °C as previously described [50].

### 4.2. Root Phenotype Analysis

For gravity response, the seeds were planted on the agar medium (1/2 MS, 0.8% Agar, 0.05% MES, 1% Sucrose, pH 5.8) and grown inside the agar on vertical plates for 7 d. The root bend formation times were counted from the moment when the plates were rotated by 90° in the clockwise direction to the time when root growth became parallel with new gravity vector [31]. For root growth pattern, the seedlings were grown for 2 d on agar-solidified minimal medium (0.25% sucrose) that was vertically set. Then, the plates were slanted at a 45° angle for 4 days [11].

### 4.3. Real-Time PCR Assay

The RNA samples were extracted from different tissues of *Arabidopsis* plants using a total RNA extraction kit (TIANGEN, Beijing, China). The cDNA templates were prepared using SuperScript II kit (Invitrogen, http://www.invitrogen.com/, accessed 2 July 2019). Using ABI 7500 real-time system (Applied Bioystems, http://www.appliedbiosystems.com/, accessed 2 August 2019) and the Power SYBR Green PCR Master Mix, all the real-time-PCR assays were performed with the primers listed in Table S3. The RNA input was normalized with the ACTIN2/8 (*At3g18780*) RNA levels. The mRNA levels were calculated with comparative Ct (threshold cycle) method as previously described [50].

### 4.4. GUS Assay for Expression Pattern

Because the *CML12* coding sequence (CDS) had three regions that shared high similarity [38], it was difficult to clone the full sequence of *CML12* CDS. For the CDS, 18 bp upstream the start codon ATG was included and the promoter sequence was from 1500 bp upstream start codon to 18 bp upstream start codon. The 1482 bp promoter fragment and 993 bp coding sequence of *CML12* were amplified using the primer pairs HindIII-PCML12/PCML12-XbaI and XbaI-TCH3/TCH3-BamHI (Table S3) from Col genomic DNA and cDNA, respectively. The resulting fragments were then inserted into the modified pCAMBIA1300 vector (CAMBIA, http://www.cambia.org/, accessed on 13 June 2020) to generate the construct p*CML12::CML12-GUS*. After being verified by sequencing, the resulting construct was introduced into Col plants using the *Agrobacterium*-mediated infiltration method [51]. GUS staining was performed as described previously [52].

### 4.5. Subcellular Localization of CML12 Protein

The 993 bp coding sequence DNA and 1482 bp promoter of *CML12* were amplified by PCR using the primer pairs HindIII-PCML12/PCML12-PstI and PstI-TCH3/TCH3-BamHI listed in Table S3. These two fragments were then inserted into the Ti-derived pCAMBIA1300 vector (CAMBIA, http://www.cambia.org/, accessed on 13 June 2020) to generate the construct p*CML12::CML12-GFP*. The construct was then introduced into *cml12-3* mutant plants. The GFP signals in the root epidermal cells were detected using the Zeiss confocal microscope (LSM710 Meta; Zeiss, http://www.zeiss.com, accessed on 21 March 2020).

### 4.6. Yeasts Two Hybrid (Y2H) Assay

The DNA fragments of *CML12* and cytoplasmic domain of *MLO4* (*MLO4C*) were amplified by PCR using the primer pairs NdeI-CML12/CML12-BamHI and NdeI-MLO4C/MLO4C-ECoRI listed in Table S3. The resulting fragments were inserted into the assay vector pairs pGADT7 (AD) and pGBKT7(BD) (Clontech, www.clontech.com/, accessed on 21 October 2020), respectively, as described previously [53]. The yeast strain AH109 was co-transformed with generated construct pairs. SD/-Leu-Trp plates were used to culture the transformed yeast cells for 3 days at 30 °C. Then, SD/-Trp-Leu-His-Ade plates were used to select the colonies with interacting protein pairs for 3 to 7 days.

### 4.7. Luciferase Complementation Imaging (LCI) Assay

The coding sequences of *CML12* and *MLO4* were amplified by PCR using the primers KpnI-CML12/CML12-SalI and KpnI-MLO4/MLO4-SalI (Table S3). These fragments were then subcloned into the vectors pCAMBIA1300-nLUC and pCAMBIA1300-cLUC, respectively. The positive control pair was SGT1-nLUC and RAR1-cLUC, which was a kind gift from Dr. Shuhua Yang (China Agricultural University, Beijing, China). Applying electroporation, all the constructs were introduced into *A. tumefaciens* strain GV3101, which was then injected into 3-week-old tobacco leaves to transiently transform the tobacco cells. Under the detection of the low-light cooled charge-coupled device camera (iKon-L936; Andor Technology, http://www.andor.com/, accessed on 21 April 2020), the fluorescence signal was detected 3–6 days after infiltration, as previously described by Chen et al. [54] and Hua et al [55].

### 4.8. Bimolecular Fluorescence Complementation (BiFC) Assays Luciferase Complementation Imaging

The coding sequences of CML12, MLO4 and MLO4C were amplified by PCR using the primers XbaI-CML12/CML12-BamHI, XbaI-MLO4/MLO4-BamHI, and XbaI-MLO4C/MLO4-BamHI (Table S2). Applying the biolistic PDS-1000/He gene gun system (Bio-Rad, http://www.biorad.com/, accessed on 11 July 2020), onion epidermal cells were transiently transformed. After cultivated on 1.5 MS culture medium (1.5% Agar, 1/2 MS) for 16–24 hours, the fluorescent signals were detected under a Leica confocal laser scanning microscope (LSM710, Carl Zeiss, http://www.zeiss.com/, accessed on 21 March 2020) as described by Gookin et al [56].

## Figures and Tables

**Table 1:** Interactive protein pairs between MLO and CaM/CML family.

MLOs	CaM/CML Proteins that Interacted with Corresponding MLO Proteins
MLO1C	CaM2, CML29
MLO2C	CML9, CML18
MLO3C	CML18, CML20, CML23, CML26, CML32, CML40, CML44
MLO4C	CML12, CML40, CML44
MLO5C	CML18, CML26, CML40, CML44
MLO6C	CML8, CML10, CML20, CML26, CML73, CML40, CML44, CML49
MLO7C	CML8, CML9, CML10, CML11, CML12, CML26, CML30, CML37, CML38, CML40, CML44
MLO8C	CML8, CML9, CML23, CML26, CML37, CML40, CML44
MLO9C	CML10, CML42, CML44, CML49
MLO10C	CML8, CML9, CML10, CML26, CML37, CML44
MLO11C	CML47
MLO12C	CML10, CML12, CML30, CML35, CML36, CML37, CML44
MLO13C	CML32, CML40, CML41
MLO14C	CML17, CML18, CML49
MLO15C	CML10, CML44

## Data Availability

We don’t report additional data.

## Supplementary Materials

The following are available online at https://www.mdpi.com/article/10.3390/ijms22115962/s1.
