# Supplementary material for: The Mildew Resistance Locus O 4 Interacts with CaM/CML and Is Involved in Root Gravity Response
Source: Int J Mol Sci. 2021 May 31;22(11):5962. doi: 10.3390/ijms22115962 (PMC8198571; doi:10.3390/ijms22115962)
Supplement: Supplementary file 1 [file ijms-22-05962-s001.zip › ijms-1234417-supplementary.pdf]

Supplemental materials

Figure S1-S8

Table S1-S3

Supplemental figure 1

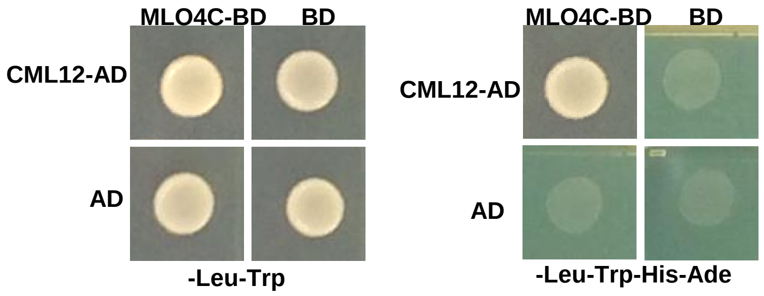

Figure S1. Y2H showed that CML12 interacted with cytoplasmic domain of MLO4 (MLO4-C).

Supplemental figure 2

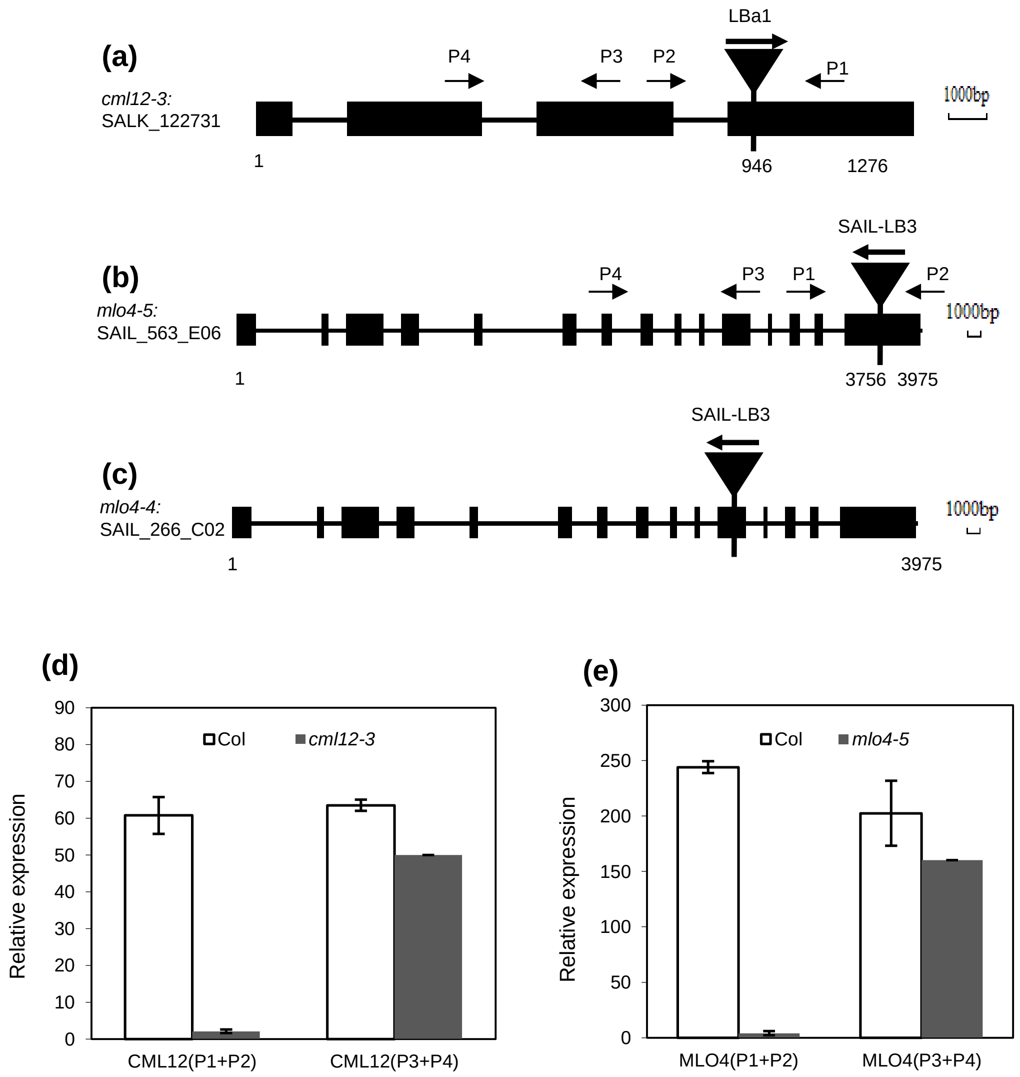

Figure S2. Identification of *cml12* and *mlo4* mutant. (a)T-DNA was inserted at the forth exon in *CML12* gene. Black box: exon; black line: intron; Arrow: primers used in the following real-time PCR. (b) T-DNA was inserted at the last exon in *MLO4* gene in *mlo4-5* mutant. (c) T-DNA was inserted at eleventh exon in *MLO4* gene in *mlo4-4* mutant. Black box: exon; black line: intron; Arrow: primers used in the following real-time PCR. (d) Real-time PCR showed that *cml12-3* was a knock-out mutant and expressed truncated protein. (E) Real-time PCR showed that *mlo4-5* was a knock-out mutant and expressed truncated protein.

Supplemental figure 3

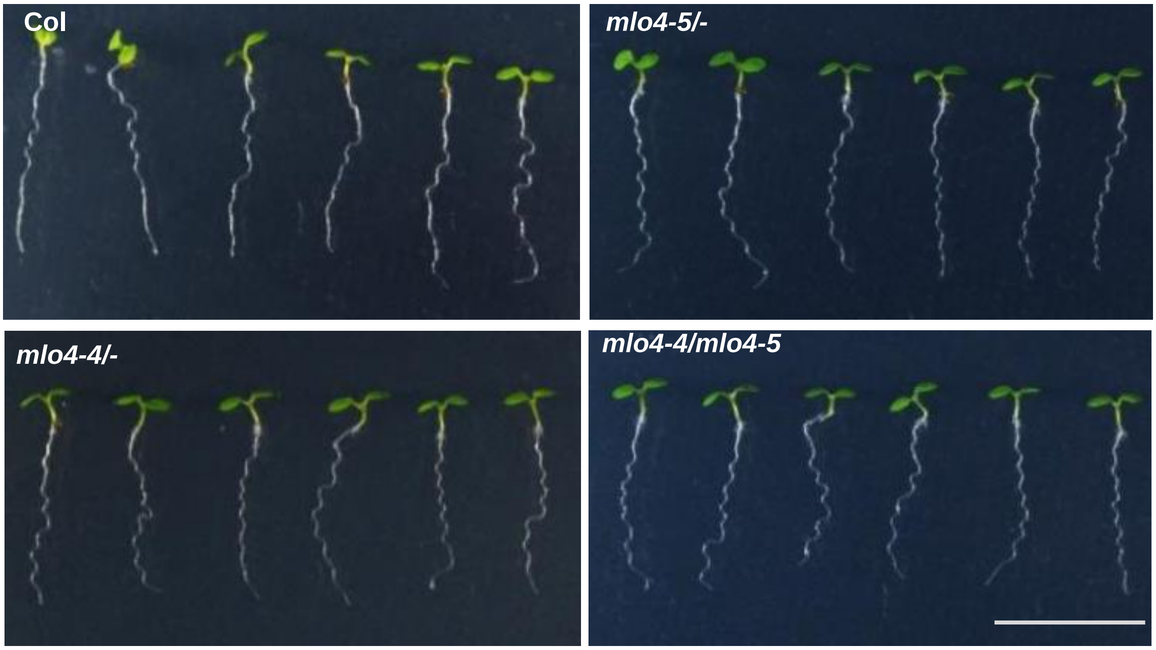

Figure S3. *mlo4-5* was another allelic mutant of *MLO4* gene.

Compared to wild-type roots, single mutant *mlo4-5* formed denser loops in roots, similar to *mlo4-4* roots. And F1 progeny of *mlo4-5* and *mlo4-4* showed resembled phenotype like both singe *mlo4* mutants. Bar = 1 cm.

Supplemental figure 4

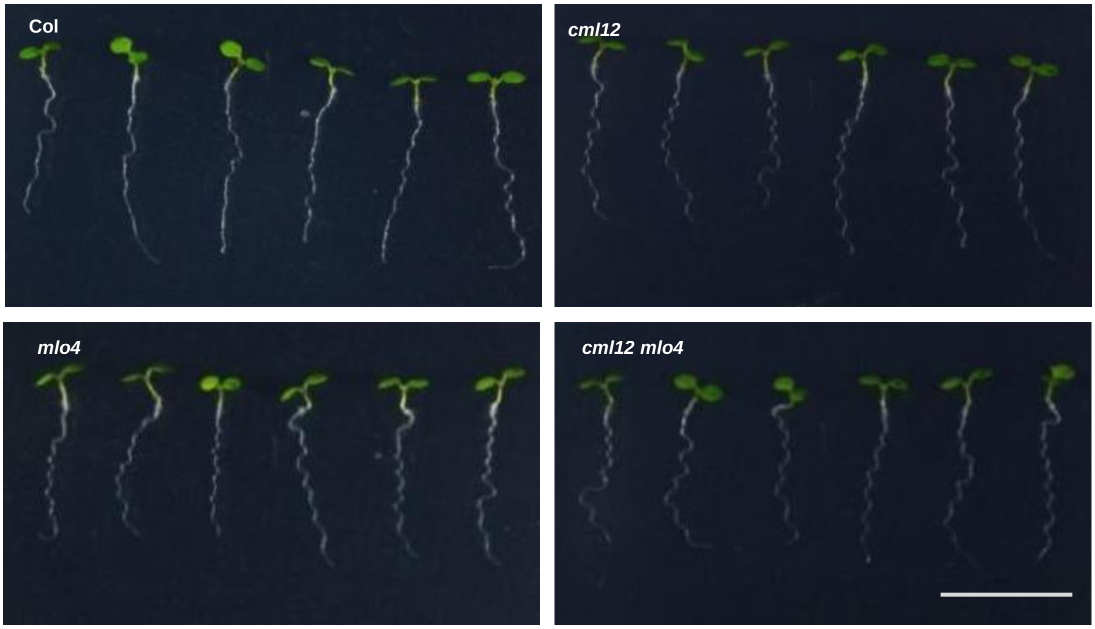

Figure S4. *cml12* and *mlo4* roots shared similar growth pattern on reclined agar medium. Single mutant *cml12* and *mlo4* roots formed denser loops and wavelengths were shorter. Phenotype of double mutant *cml12 mlo4* wasn't obviously aggravated. Bar = 1 cm.

Supplemental figure 5

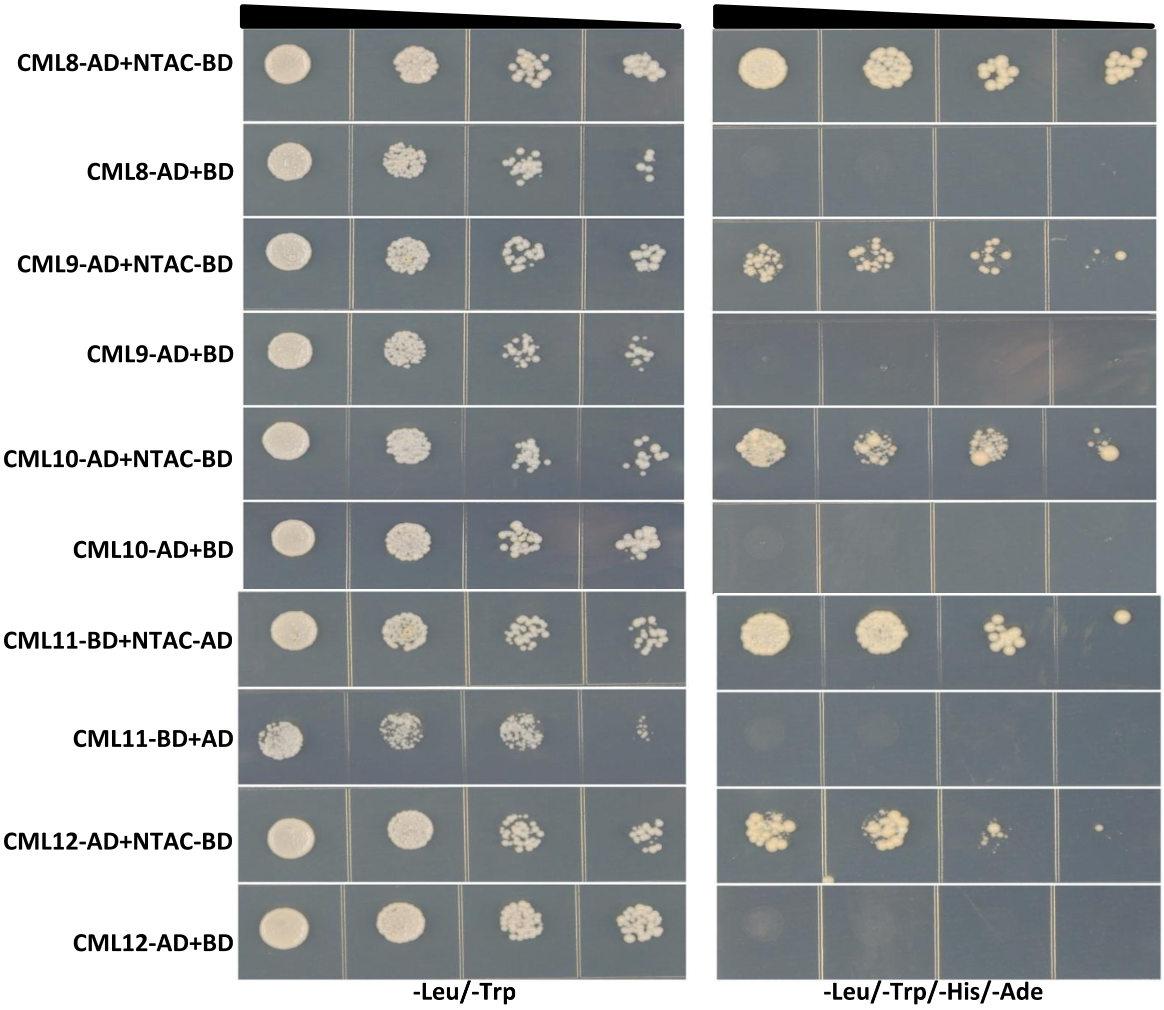

Figure S5. Y2H showed that NTA C-termini (NTAC) interacted with CML8-12 in yeast cells.

Supplemental figure 6

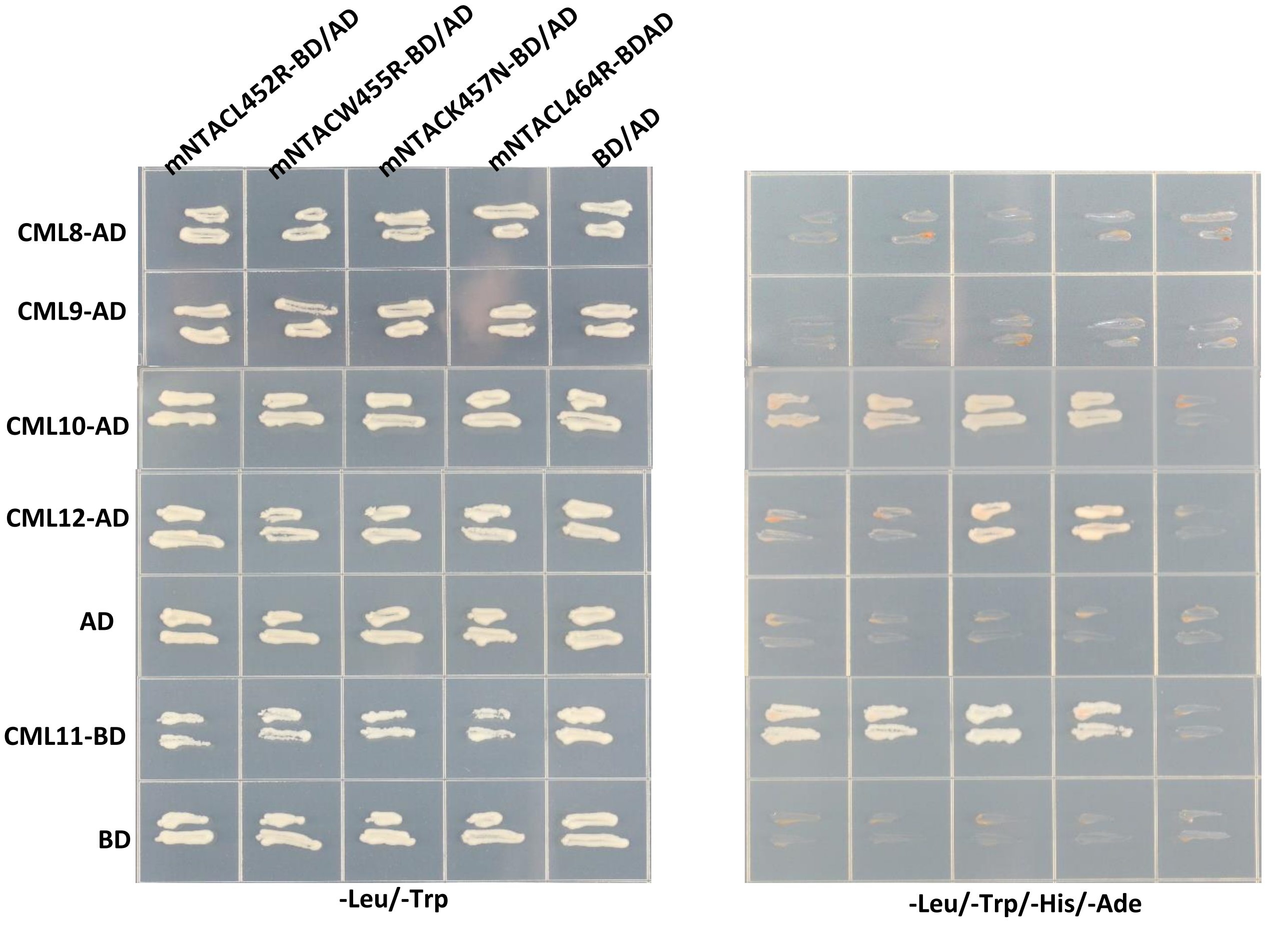

Figure S6. Y2H showed interaction between NTA and CMLs was specific.
NTA variants (L452R, W455R) with conservative sites changed were unable to interact with CML12. And NTA variants (K457N, L464R) with unconservative sites changed were still able to interact with CML12. All NTA variants (L452R, W455R, K457N, L464R) failed to interact with CML8 or CML9 but still bound CML10 and CML11.

Supplemental figure 7

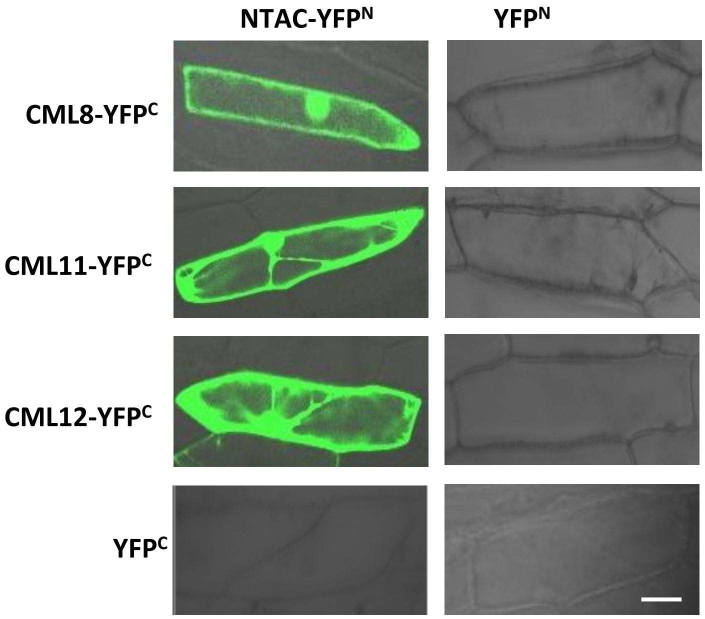

Figure S7. BiFC showed that cytoplasmic domain of NTA interacted with CML8, CML9 and CML11.Bars= 50 μm.

Supplemental figure 8

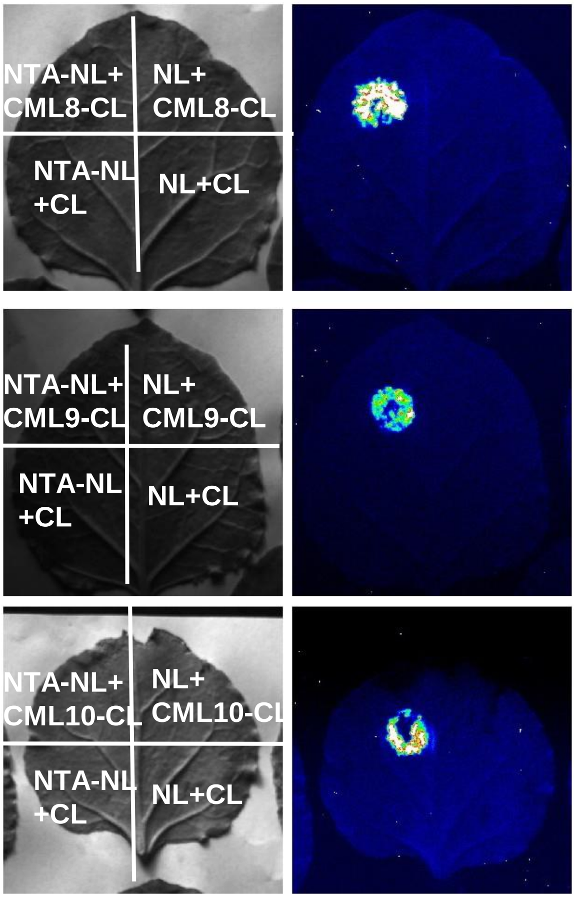

Figure S8. LCI showed full length of MLO4 interacted with CML8, CML9 and CML10 in tobacco cells.

Table S1. CML-BD interacted with MLO-AD

√ 互作	× 不互作	* 自激活	* 无自激活

	AD	MLO1C-AD	MLO2C-AD	MLO3C-AD	MLO4C-AD	MLO5C-AD	MLO6C-AD	MLO7C-AD	MLO8C-AD	MLO9C-AD	MLO10C-AD	MLO11C-AD	MLO12C-AD	MLO13C-AD	MLO14C-AD	MLO15C-AD
BD	*	*	*	*	*	*	*	*	*	*	*	*	*	*	*	*
CaM1-BD	*	×	×	×	×	×	×	×	×	×	×	×	—	×	×	×
CaM2-BD	*	√	×	×	×	×	×	×	×	×	×	×	—	×	×	×
CaM3-BD	*	×	×	×	×	×	×	×	×	×	×	×	—	×	×	×
CaM4-BD	*	×	×	×	×	×	×	×	×	×	×	×	—	×	×	×
CaM5-BD	*	×	×	×	×	×	×	×	×	×	×	×	—	×	×	×
CaM6-BD	*	×	×	×	×	×	×	×	×	×	×	×	—	×	×	×
CaM7-BD	*	×	×	×	×	×	×	×	×	×	×	×	—	×	×	×
CML1-BD	*	×	×	×	×	×	×	×	×	×	×	×	—	×	×	×
CML2-BD	*	—	—	—	—	—	—	—	—	—	—	—	—	—	—	—
CML3-BD	*	×	×	×	×	×	×	×	×	×	×	×	—	×	×	×
CML4-BD	*	×	×	×	×	×	×	×	×	×	×	×	—	×	×	×
CML5-BD	*	×	×	×	×	×	×	×	×	×	×	×	—	×	×	×
CML6-BD	*	×	×	×	×	×	×	×	×	×	×	×	—	×	×	×
CML7-BD	*	×	×	×	×	×	×	×	×	×	×	×	—	×	×	×
CML8-BD	*	—	—	—	—	—	—	—	—	—	—	—	—	—	—	—
CML9-BD	*	—	—	—	—	—	—	—	—	—	—	—	—	—	—	—
CML10-BD	*	×	×	×	×	×	×	×	×	√	√	×	—	×	×	×
CML11-BD	*	—	—	—	—	—	—	—	—	—	—	—	—	—	—	—
CML12-BD	*	×	×	×	×	×	×	√	×	×	×	×	—	×	×	×
CML13-BD	*	×	×	×	×	×	×	×	×	×	×	×	—	×	×	×
CML14-BD	*	×	×	×	×	×	×	×	×	×	×	×	—	×	×	×
CML15-BD	*	×	×	×	×	×	×	×	×	×	×	×	—	×	×	×
CML16-BD	*	×	×	×	×	×	×	×	×	×	×	×	—	×	×	×
CML17-BD	*	×	×	×	×	×	×	×	√	√	√	×	—	×	√	×
CML18-BD	*	×	×	√	×	√	×	×	×	×	×	×	—	×	√	×
CML19-BD	*	—	—	—	—	—	—	—	—	—	—	—	—	—	—	—
CML20-BD	*	×	×	√	×	×	√	×	×	√	√	√	—	×	√	×
CML21-BD	*	×	×	×	×	×	×	×	×	×	×	×	—	×	×	×
CML22-BD	*	×	×	×	×	×	×	×	×	×	×	×	—	×	×	×
CML23-BD	*	×	×	√	×	×	×	×	√	×	×	×	—	×	×	×
CML24-BD	*	×	×	×	×	×	×	×	×	×	×	×	—	×	×	×
CML25-BD	*	×	×	×	×	×	×	×	×	×	×	×	—	×	×	×
CML26-BD	*	×	×	√	×	√	√	√	√	×	√	×	—	×	×	×
CML27-BD	*	×	×	×	×	×	×	×	×	×	×	×	—	×	×	×
CML28-BD	*	×	×	×	×	×	×	×	×	×	×	×	—	×	×	×
CML29-BD	*	×	×	×	×	×	×	×	×	×	×	×	—	×	×	×
CML30-BD	*	×	×	×	×	×	×	×	×	×	×	×	—	×	×	×
CML31-BD	*	×	×	×	×	×	×	×	×	×	×	×	—	×	×	×
CML32-BD	*	×	×	√	√	√	√	√	√	√	√	×	—	√	×	×
CML33-BD	*	×	×	×	×	×	√	×	×	×	×	×	—	×	×	×
CML34-BD	*	—	—	—	—	—	—	—	—	—	—	—	—	—	—	—
CML35-BD	*	—	—	—	—	—	—	—	—	—	—	—	—	—	—	—
CML36-BD	*	—	—	—	—	—	—	—	—	—	—	—	—	—	—	—
CML37-BD	*	—	—	—	—	—	—	—	—	—	—	—	—	—	—	—
CML38-BD	*	—	—	—	—	—	—	—	—	—	—	—	—	—	—	—
CML39-BD	*	×	×	×	×	×	×	×	×	×	×	×	—	×	×	×
CML40-BD	*	×	×	√	√	√	√	√	√	√	√	×	—	√	×	×
CML41-BD	*	×	×	×	×	×	×	×	×	×	×	×	—	√	×	×
CML42-BD	*	×	×	×	×	×	×	×	×	√	×	×	—	×	×	×
CML43-BD	*	×	×	×	×	×	×	×	×	×	×	×	—	×	×	×
CML44-BD	*	×	×	√	√	√	√	√	√	√	√	×	—	×	×	√
CML45-BD	*	×	×	×	×	×	×	×	×	×	×	×	—	×	×	
CML46-BD	*	×	×	×	×	×	×	×	×	×	×	×	—	×	×	×
CML47-BD	*	×	×	×	×	×	×	×	×	×	×	√	—	×	×	×
CML48-BD	*	—	—	—	—	—	—	—	—	—	—	—	—	—	—	—
CML49-BD	*	×	×	×	×	×	√	×	×	√	×	×	—	√	×	×
CML50-BD	*	—	—	—	—	—	—	—	—	—	—	—	—	—	—	—

To be continued in the next page

Table S2. CML-AD interacted with MLO-BD

	BD	MLO1C-BD	MLO2C-BD	MLO3C-BD	MLO4C-BD	MLO5C-BD	MLO6C-BD	MLO7C-BD	MLO8C-BD	MLO9C-BD	MLO10C-BD	MLO11C-BD	MLO12C-BD	MLO13C-BD	MLO14C-BD	MLO15C-BD
AD	*	*	*	*	*	*	*	*	*	*	*	*	*	*	*	*
CaM1-AD	*	×	×	×	—	×	×	×	×	×	×	×	×	×	×	×
CaM2-AD	*	×	×	×	—	×	×	×	×	×	×	×	×	×	×	×
CaM3-AD	*	×	×	×	—	×	×	×	×	×	×	×	×	×	×	×
CaM4-AD	*	×	×	×	—	×	×	×	×	×	×	×	×	×	×	×
CaM5-AD	*	×	×	×	—	×	×	×	×	×	×	×	×	×	×	×
CaM6-AD	*	×	×	×	—	×	×	×	×	×	×	×	×	×	×	×
CaM7-AD	*	×	×	×	—	×	×	×	×	×	×	×	×	×	×	×
CML1-AD	*	×	×	×	—	×	×	×	×	×	×	×	×	×	×	×
CML2-AD	*	×	×	×	—	×	×	×	×	×	×	×	×	×	×	×
CML3-AD	*	×	×	×	—	×	×	×	×	×	×	×	×	×	×	×
CML4-AD	*	×	×	×	—	×	×	×	×	×	×	×	×	×	×	×
CML5-AD	*	×	×	×	—	×	×	×	×	×	×	×	×	×	×	×
CML6-AD	*	×	×	×	—	×	×	×	×	×	×	×	×	×	×	×
CML7-AD	*	×	×	×	—	×	×	×	×	×	×	×	×	×	×	×
CML8-AD	*	×	×	×	—	×	√	√	√	×	√	×	×	×	×	×
CML9-AD	*	×	√	×	—	×	×	×	√	×	√	×	×	×	×	×
CML10-AD	*	×	×	×	—	×	√	√	×	√	√	×	√	×	×	√
CML11-AD	*	×	×	×	—	×	×	√	×	×	×	×	×	×	×	×
CML12-AD	*	×	×	×	√	×	×	√	×	×	×	×	√	×	×	×
CML13-AD	*	×	×	×	—	×	×	×	×	×	×	×	×	×	×	×
CML14-AD	*	×	×	×	—	×	×	×	×	×	×	×	×	×	×	×
CML15-AD	*	×	×	×	—	×	×	×	×	×	×	×	×	×	×	×
CML16-AD	*	×	×	×	—	×	×	×	×	×	×	×	×	×	×	×
CML17-AD	*	×	×	×	—	×	×	×	×	×	×	×	×	×	×	×
CML18-AD	*	×	×	×	—	×	×	×	×	×	×	×	×	×	×	×
CML19-AD	*	×	×	×	—	×	×	×	×	×	×	×	×	×	×	×
CML20-AD	*	×	×	×	—	×	×	×	×	×	×	×	×	×	×	×
CML21-AD	*	×	×	×	—	×	×	×	×	×	×	×	×	×	×	×
CML22-AD	*	×	×	×	—	×	×	×	×	×	×	×	×	×	×	×
CML23-AD	*	×	×	×	—	×	×	×	×	×	×	×	×	×	×	×
CML24-AD	*	×	×	×	—	×	×	×	×	×	×	×	×	×	×	×
CML25-AD	*	×	×	×	—	×	×	×	×	×	×	×	×	×	×	×
CML26-AD	*	×	×	×	—	×	×	×	×	×	×	×	×	×	×	×
CML27-AD	*	×	×	×	—	×	×	×	×	×	×	×	×	×	×	×
CML28-AD	*	×	×	×	—	×	×	×	×	×	×	×	×	×	×	×
CML29-AD	*	×	√	×	—	×	×	×	×	×	×	×	×	×	×	×
CML30-AD	*	×	×	×	—	×	×	×	×	×	×	×	√	×	×	×
CML31-AD	*	×	×	×	—	×	×	×	×	×	×	×	×	×	×	×
CML32-AD	*	×	×	×	—	×	×	×	×	×	×	×	×	×	×	×
CML33-AD	*	×	×	×	—	×	×	×	×	×	×	×	×	×	×	×
CML34-AD	*	×	×	×	—	×	×	×	×	×	×	×	×	×	×	×
CML35-AD	*	×	×	×	—	×	×	×	×	×	×	×	√	×	×	×
CML36-AD	*	×	×	×	—	×	×	×	×	×	×	×	√	×	×	×
CML37-AD	*	×	×	×	—	×	√	√	×	×	√	×	√	×	×	×
CML38-AD	*	×	×	×	—	×	×	√	×	×	×	×	×	×	×	×
CML39-AD	*	×	×	×	—	×	×	×	×	×	×	×	×	×	×	×
CML40-AD	*	×	×	×	—	×	×	×	×	×	×	×	×	×	×	×
CML41-AD	*	×	×	×	—	×	×	×	×	×	×	×	×	×	×	×
CML42-AD	*	×	×	×	—	×	×	×	×	×	×	×	×	×	×	×
CML43-AD	*	×	×	×	—	×	×	×	×	×	×	×	×	×	×	×
CML44-AD	*	×	×	×	—	×	×	×	×	×	×	×	√	×	×	×
CML45-AD	*	×	×	×	—	×	×	×	×	×	×	×	×	×	×	×
CML46-AD	*	×	×	×	—	×	×	×	×	×	×	×	×	×	×	×
CML47-AD	*	×	×	×	—	×	×	×	×	×	×	×	×	×	×	×
CML48-AD	*	×	×	×	—	×	×	×	×	×	×	×	×	×	×	×
CML49-AD	*	×	×	×	—	×	×	×	×	×	×	×	×	×	×	×
CML50-AD	*	×	×	×	—	×	×	×	×	×	×	×	×	×	×	×

Table S3. Primers used in the study.

Uses of the primers	Names of the primers	Sequences of the primers (5'-3')
Identify mutants	SAIL-LB3	TAGCATCTGAATTTCATAACCAATCTCGATACAC
	SAIL266C02-1	ATCTCGTTCCAATCCATGCTA
	SAIL266C02-2	CTCATCTTCCATCGTCCGTA
	SAIL563E06-1	GTTCTTGgtaaggccaccttataaaatgca
	SAIL563E06-2	cctaatgccataatatctcgttccaatccatgc
	LBa1	TGGTTCACGTAGTGGGCCATCG
	SK122731-1	ggtaacacactaacacatgc
	SK122731-2	AGGTCATGATCAAGCGCC
Real-time-PCR	MLO4-P1	GAGACTCACTTCACAGTTGG
	MLO4-P2	CTCTGCTTGGTCTATGGAGT
	MLO4-P3	GGCAACATTCATCTGGTTCT
	MLO4-P4	CCATAACTACACCAGAACTG
	CML12-P1	GTACTACTATGCGCTCCCTT
	CML12-P2	GACCTTGATTCACAACTCTG
	CML12-P3	TACCGTGATGTTTTCCCTCG
	CML12-P4	ATCTGATCGTCAGTGAGCTG
GUS assay	HindIII-PCML12	CCC AAGCTT AATGACCGACTTCATCAAAA
	PCML12-XbaI	AGC TCTAGA tgatgactgaaagaagagtttgtga
	XbaI-TCH3	AGC TCTAGA CAGAAAACAAAAAAAACA
	TCH3-BamHI	TCA GGATCC AGATAACAGCGCTTCGAACA
Complementation	HindIII-PCML12	CCC AAGCTT AATGACCGACTTCATCAAAA
	PCML12-PstI	CGA CTGCAG tgatgactgaaagaagagtttgtga
	PstI-TCH3	CGA CTGCAG CAGAAAACAAAAAAAACA
	TCH3-BamHI	TCA GGATCC AGATAACAGCGCTTCGAACA
Y2H	NdeI-CML12	CTC CATATG CAGAAAACAAAAAAAACAAT
	CML12-BamHI	CTA GGATCC AGATAACAGCGCTTCGAACA
	NdeI-MLO4C	CTC CATATG ACTCAGATGGGATCTCGGCAT
	MLO4C-ECoRI	CTG GAATTC AGTCCTCCTAAACAACTCA

To be continued in the next page

BiFC	XbaI-CML12	AGC TCTAGA CAGAAAACAAAAAAAACA
	CML12-BamHI	TCA GGATCC AGATAACAGCGCTTCGAACA
	XbaI-MLO4	CTG TCTAGA ATGGAGCATATGATGAAAGAAGG
	MLO4-BamHI	CTA GGATCC AGTCCTCCTAAACAACTCAAG
	XbaI-MLO4C	TAG TCTAGA ACTCAGATGGGATCTCGGCA
LCI	KpnI-CML12	TAC GGTACC CAGAAAACAAAAAAAACA
	CML12-SalI	CAT GTCGAC TCAAGATAACAGCGCTTCGAACAA
	KpnI-MLO4	CTA GGTACC ATGGAGCATATGATGAAAGAAGG
	MLO4-SalI	CTA GTCGAC AGTCCTCCTAAACAACTCAAG
Y2H	NcoI-CaM1	CATG CCATGG AC ATGGCGGATC AACTCACTGA
	CaM1-SalI	CGC GTCGAC CTTAGCCATCATAATCTTGA
	CAM1-CLaI	CCC ATCGAT AC ATGGCGGATC AACTCACTGA
	NdeI-CaM2	ATC CATATG ATGGCGGATCAGCTCACAGACGATCAG
	CaM2-BamHI	CAT GGATCC CTTAGCCATCATAACCTTCA
	ECoRI-CaM3	CTC GAATTC ATGGCGGATCAGCTCACCGA
	CaM3-BamHI	CGC GGATCC CTTAGCCATCATGACCTTAA
	CAM4-ECoRI	CCG GAATT ATGATTAGCT AAAAACCCAA CTAG
	CAM4-BamHI	CGC GGATCC CAACAAAATA AAAAAAGATA AGTC
	NdeI-CaM5	CGA CATATG ATGGCAGATCAGCTCACCGATGATC
	CaM5-BamHI	CGA GGATCC GAGAATACGGCAGTGACTTTT
	BamHI-CaM6	CGC GGATCC AC ATGGCGGATCAGCTCACCGATGACCAGA
	CaM6-XhoI	ATC CTCGAG CTTAGCCATCATGACTTTGA
	CaM6-SalI	CGC GTCGAC CTTAGCCATCATGACTTTGACGAATTC
	ECoRI-CaM7	CGC GAATTC ATGGCGGATCAGCTAACCGA
	CaM7-BamHI	CCG GGATCC CTTTGCCATCATGACTTTGA
	ECoRI-CML1	CAT GAATTC ATGCCGATAT TCCAGTGGCT
	CML1-BamHI	CAC GGATCC TCTATTGCTA AAGTCACCAGT

To be continued in the next page

Y2H	ECoRI-CML2	CCG GAATTC ATGGATCGTG GAGAATTGAGT AGAG
	CML2-BamHI	CGC GGATCC ATTGGAGCTA AGAGCAGCAAA TCC
	ECoRI-CML3	CCG GAATTC ATGGATCAAG CGGAGCTTGCC AGGAT
	CML3-BamHI	CGC GGATCC CAAGTTTGAT CCTAAGGCGGC A
	NdeI-CML4	CGC CATATG ATGGTGAGAG TCTTTCTTCTC
	CML4-BamHI	CGC GGATCC TGATCTATTG CTAAAGTCACC AC
	ECoRI-CML5	CCG GAATTC ATGGTGAGAA TATTCCTTCTC TAC
	CML5-BamHI	CGC GGATCC ATTACTGCTG CTAAAGCCACC ACC
	ECoRI-CML6	CCG GAATTC ATGGATTCCA CGGAGCTGAAC CGT
	CML6-BamHI	CGC GGATCC GCTCAAAGAG CTAAAAAAGCG ACCC
	NdeI-CML7	CGC CATATG ATGGATCCGA CAGAGCTAAAA CGCGTGTTC
	CML7-ECoRI	ATC GAATTC CAAAGAATTA AAACCACCGCC TTTC
	ECoRI-CML8	CCG GAATTC ATGGAAGAAA CAGCACTGAC AA
	CML8-BamHI	CGC GGATCC GTCAATGTTG ATCATCATCT TG
	NcoI-CML9	CTA CCATTG AG ATGGCGGATGCTTTCACAGATG
	ClaI-CML9	CCC ATCGAT AC ATGGCGGATG CTTTCACAGA TGAAC
	CML9-BamHI	CGC GGATCC ATAAGAGGCA GCAATCATCA TT
	ECoRI-CML10	CCG GAATTC ATGGCGAATA AGTTCACTAG ACAA
	CML10-BamHI	CGC GGATCC AGAAAACAAC GCTTCGAACA AA
	ECoRI-CML11	CCG GAATTC ATGGAAGAAA TTCAACAACA ACAAC
	CML11-BamHI	CGC GGATCC ACCATTGATC ATCATCATCC TGAC
	NdeI-CML13	CGC CATATG ATGGGGAAAG ATGGTCTGAG CGAC
	CML13-ECoRI	CCG GAATTC CTTAGCAACC ATCCTTGCTA TG
	ECoRI-CML14	CCG GAATTC ATGAGCAAGG ATGGTTTGAG CA
	CML14-BamHI	CGC GGATCC TTACTTAGCA ACCATTCTAG C
	NdeI-CML15	CGC CATATG ATGGAGGATC AGATAAGACA AC
	CML15-BamHI	CGC GGATCC CGAATTAATT TTTAATCCAA AGTAATC
	NdeI-CML16	CGC CATATG ATGGCGTCAA CAAAACCAAC CGAT
	CML16-ECoRI	CCG GAATTC AGAAGCGGTT AATCCAAGAA
	ECoRI-CML17	CCG GAATTC ATGAGTCACA AAGTCTCCAA AAAG
	CML17-BamHI	CGC GGATCC ACCCCATATA TCATCAAATG CAGC

To be continued in the next page

Y2H	ECoRI-CML18	CCG GAATTC ATGAGCTGCG ACGGAGGCAA A
	CML18-BamHI	CGC GGATCC ACCCCAAGCA TTATCAAACG CAGCT
	ECoRI-CML19	CCG GAATTC ATGGCGAATT ACATGTCGGA AGCAG
	CML19-BamHI	CGC GGATCC GCCGTAAGAG GTTCTCTTCA TCATC
	NcoI-CML20	CATG CCATGG AC ATGTCGAGTA TATACAGAAC TG
	CML20-CLaI	CCC ATCGAT AC ATGTCGAGTA TATACAGAAC TG
	CML20-BamHI	CGC GGATCC CTAGTTACCA CCATAAGCAG TTC
	NdeI-CML21	CGC CATATG ATGGGAGGTGCAGTGACGAAATCTGA
	CML21-BamHI	CGC GGATCC AGCTTTCTCATTGTTGTCTTC
	ECoRI-CML22	CCG GAATTC ATGCTTTGCTGCTGTGTTAACT
	CML22-BamHI	CGC GGATCC GCTGCTCATAAAATCATCATCTGC
	ECoRI-CML23	CCG GAATTC ATGTCGAAGAACGTTTCGAGAAAC
	CML23-BamHI	CGC GGATCC AGCACTACCATTAATCATCATCAT
	NcoI-CML24	CATG CCATGG AC ATGTCATCGA AGAACGGAGT TG
	CML24-CLaI	CCC ATCGAT AC ATGTCATCGA AGAACGGAGT TG
	CML24-BamHI	CGC GGATCC TCAAGCACCA CCACCATTAC TC
	NdeI-CML25	CGC CATATG ATGTTCAACAAAAACCAAGGATCC
	CML25-ECoRI	ATC GAATTC CCTCGGACCACCTCCCATGACAT
	ECoRI-CML26	CCG GAATTC ATGGCAAACACAAATCTTGAATCCA
	CML26-BamHI	CGC GGATCC AGAGTTCGCAACGGTTCCCTTAACG
	NcoI-CML27	CATG CCATGG AC ATGGCAAGCG CGAATCCAGA AACC
	CML27-CLaI	CCC ATCGAT AC ATGGCAAGCG CGAATCCAGA AACC
	CML27-SacI	C GAGCT CTAGGTCGAA GGAGGAGCGG CGGAT
	NdeI-CML28	CGC CATATG ATGGCTGACGCAACAGAAAAAGCTG
	CML28-BamHI	CTA GGATCC GAAAATCTTGGCAACGTCTTTC
	NdeI-CML29	CAT CATATG ATGGCTGATGCAACGGAGAAAGCCG
	CML29-BamHI	CTC GGATCC GAAAATTTTGGCAACATCCTTCATAAGTCC
	ECoRI-CML30	CCG GAATTC ATGTCAAACGTGAGTTTTCTTGAGTTGC
	CML30-BamHI	CGC GGATCC GACATTGTTGGAAGACATCATTTTG
	NdeI-CML31	CGC CATATG ATGGCAGAGATTTTCGAAAGTGTTGAC
	CML31-BamHI	CGC GGATCC ATGTGACTCTTTGTTAGAGTTCATC
	ECoRI-CML32	CCG GAATTC ATGTCTGTTGCAGAGATCTTCGAG
	CML32-BamHI	CGC GGATCC TTGTAATTTTTTGTTGTTACAACTCATC

To be continued in the next page

Y2H	ECoRI-CML33	CCG GAATTC ATGAATAATATGTCTTTAAGTGATATC
	CML33-BamHI	CGC GGATCC AACTCCTACGAATCTAATATAACC
	ECoRI-CML34	CCG GAATTC ATGTCTGCAAAACGTGTCTTCGAG
	CML34-BamHI	CGC GGATCC TATGTCACCAATAACCAAAGCCAT
	ECoRI-CML35	CCG GAATT ATGAAGCTCG CCGCTAGCCT CAA
	CML35-BamHI	CGC GGATCC CTAATGATGA TGATCATTCA TCGC
	MLO2C-ECoRI	CTC GAATTC TTTCTTAAAAGAAAAATCTC
	NdeI-MLO3C	CTC CATATG ACTCAGATGGGGTCAAGCTACAA
	MLO3C-ECoRI	CTG GAATTC CCTTTCAGTTTTCTCTTGTA
	NdeI-MLO4C	CTC CATATG ACTCAGATGGGATCTCGGCAT
	MLO4C-ECoRI	CTG GAATTC AGTCCTCCTAAACAACTCA
	NdeI-MLO5C	CTC CATATG ACACTTCCTCTTTACGCTCTCG
	MLO5C-ECoRI	CTA GAATTC GGGACCGCTTAAGAGGTCT
	NdeI-MLO6C	CTC CATATG GTTACTCAAATGGGTTCAAAG
	MLO6C-SacI	TCA GAGCTC TCGCTTAAACGAAAAATCCC
	MLO6C-SalI	TCA GTCGAC TCGCTTAAACGAAAAATCCC
	NdeI-MLO8C	CTC CATATG ACTCAGATGGGTTCTCGGATG
	MLO8C-ECoRI	CCG GAATTC CCGGTCTTGAGATGATTCA
	NdeI-MLO9C	CTC CATATG ACATTGCCCCTTTACGCTCT
	MLO9C-ECoRI	CTG GAATTC CTTTTCATTCTTTTTCCTCGCC
	NdeI-MLO10C	CTC CATATG CAGATGGGTTCAAACATGAAG
	MLO10C-ECoRI	CTG GAATTC GTCAATATCATTAGCAGGAACG
	NdeI-MLO11C	CTC CATATG ACTCAGATGGGAACGAACTAT
	MLO11C-ECoRI	CTG GAATTC GACTCTCTTCTCACTTGGCA
	NdeI-MLO12C	TCA CATATG GTTACTCAGATGGGAACATCA
	MLO12C-BamHI	TCA GGATCC CTTCTTGAACGTAAACTCAGAC
	NdeI-MLO13C	CTC CATATG ACACAAATGGGTAGCAAATTC
	MLO13C-ECoRI	CTG GAATTC AGGGTTTTCACTTTGGACA
	NdeI-MLO14C	CTC CATATG ACTCAGATGGGAACAAACTAC
	MLO14C-ECoRI	CTG GAATTC ACATTCTCTTCTCATTGGC
	NdeI-MLO15C	CTC CATATG CTCGTCATCGGGGTAATAAT
	MLO15C-ECoRI	CTG GAATTC ATCATGGTGAGCAATCTCTGAT
	CML48-BamHI	CGC GGATCC ATCATAGGTGGCGATGAACGGAATG

To be continued in the next page

Y2H	ECoRI-CML49	CTA GAATTC ATGTCTGGTTATCCTCCATC
	CML49-BamHI	ATC GGATCC AGCGACGAGGAATGGTAAAAC
	ECoRI-CML50	CCG GAATTC ATGTCAGGATATCCTCCGACTAGCCA
	CML50-BamHI	CGC GGATCC TGCGATGAGGAAGGGGAGGACGGT
	NdeI-MLO1C	CTC CATATG TCACAGATGGGAAGTAGCTTCAAG
	MLO1C-BamHI	TCA GGATCC GTTGTTATGATCAGGTGTAATCTC
	NdeI-MLO2C	CTC CATATG ACTCAGATGGGTAGTAAAATG
	MLO2C-ECoRI	CTC GAATTC TTTCTTAAAAGAAAAATCTC
	NdeI-MLO3C	CTC CATATG ACTCAGATGGGGTCAAGCTACAA
	MLO3C-ECoRI	CTG GAATTC CCTTTCAGTTTTCTCTTGTA
	NdeI-MLO4C	CTC CATATG ACTCAGATGGGATCTCGGCAT
	MLO4C-ECoRI	CTG GAATTC AGTCCTCCTAAACAACTCA
	NdeI-MLO5C	CTC CATATG ACACTTCCTCTTTACGCTCTCG
	MLO5C-ECoRI	CTA GAATTC GGGACCGCTTAAGAGGTCT
	NdeI-MLO6C	CTC CATATG GTTACTCAAATGGGTTCAAAG
	MLO6C-SacI	TCA GAGCTC TCGCTTAAACGAAAAATCCC
	MLO6C-SalI	TCA GTCGAC TCGCTTAAACGAAAAATCCC
	NdeI-MLO8C	CTC CATATG ACTCAGATGGGTTCTCGGATG
	MLO8C-ECoRI	CCG GAATTC CCGGTCTTGAGATGATTCA
	NdeI-MLO9C	CTC CATATG ACATTGCCCCTTTACGCTCT
	MLO9C-ECoRI	CTG GAATTC CTTTTCATTCTTTTTCCTCGCC
	NdeI-MLO10C	CTC CATATG CAGATGGGTTCAAACATGAAG
	MLO10C-ECoRI	CTG GAATTC GTCAATATCATTAGCAGGAACG
	NdeI-MLO11C	CTC CATATG ACTCAGATGGGAACGAACTAT
	MLO11C-ECoRI	CTG GAATTC GACTCTCTTCTCACTTGGCA
	NdeI-MLO12C	TCA CATATG GTTACTCAGATGGGAACATCA
	MLO12C-BamHI	TCA GGATCC CTTCTTGAACGTAAACTCAGAC
	NdeI-MLO13C	CTC CATATG ACACAAATGGGTAGCAAATTC
	MLO13C-ECoRI	CTG GAATTC AGGGTTTTCACTTTGGACA
	NdeI-MLO14C	CTC CATATG ACTCAGATGGGAACAAACTAC
	MLO14C-ECoRI	CTG GAATTC ACATTCTCTTCTCATTGGC
	NdeI-MLO15C	CTC CATATG CTCGTCATCGGGGTAATAAT
	MLO15C-ECoRI	CTG GAATTC ATCATGGTGAGCAATCTCTGAT
